# Live-bearing without placenta: Physical estimation indicates the high oxygen-supplying ability of white shark uterus to the embryo

**DOI:** 10.1038/s41598-017-11973-9

**Published:** 2017-09-18

**Authors:** Taketeru Tomita, Ryo Nozu, Masaru Nakamura, Shohei Matsuzaki, Kei Miyamoto, Keiichi Sato

**Affiliations:** 1 Zoological Laboratory, Okinawa Churashima Research Center, 888 Ishikawa, Motobu-cho, Okinawa, 905-0206 Japan; 2 Okinawa Churaumi Aquarium, 424 Ishikawa, Motobu-cho, Okinawa, 905-0206 Japan

## Abstract

One of the mysteries of shark aplacental viviparity is the ability of the embryos to acquire oxygen from their mothers without a placental connection. It has been assumed that embryonic respiration in aplacental viviparous shark depends on oxygen from the uterine wall, although this hypothesis has not been confirmed quantitatively. Morphological observations of the uterine wall of white shark (*Carcharodon carcharias*) provided the first quantitative evidence to support the ability of the uterus to supply ample oxygen to the embryo of viviparous elasmobranchs. The uterine surface of the white shark is characterized by (1) uterine lamellae that develop perpendicular to the uterine wall, (2) uterine lamellae folded in an accordion-like fashion, and (3) numerous micro-ridges on the lamellar surface. These modifications result in increased uterine surface are to up to 56 folds compared to the uterus with a smooth surface. Histological observations revealed that the diffusion barrier of the uterine wall is approximately 12 µm. By using these values, the oxygen-diffusion capacity of 1 cm^2^ of the uterine wall of white shark was estimated to be 63.6 nmol·min^−1^·torr^−1^. This value is 250–400 times greater than that observed in other aplacental viviparous sharks (*Squalus* spp.) and is comparable with that of fish gills.

## Introduction

Oxygen acquisition is essential for all living vertebrates, including the embryos developing in the maternal body. Therefore, viviparous (live-bearing) vertebrates have acquired various methods to supply oxygen to the embryo in utero. It has been assumed that some carcharhiniform sharks and mammals present convergent evolution of embryonic respiratory mechanism: The embryos/fetuses are connected to the uterus by a placenta and they acquire oxygen from the uterus through this connection^1^. However, the development of the placenta is restricted to few clades of vertebrates, and the mechanism of oxygen delivery to embryo in aplacental vertebrates, including most viviparous elasmobranchs (sharks and batoids), is still largely unknown^2^.

It is widely believed that embryonic respiration in aplacental elasmobranchs depends on the oxygen diffused from the uterine wall^3–5^. This hypothesis is supported by the presence of numerous surface projections (e.g., trophonemata and uterine villi) on the inner wall of the uterus, which increase the surface area for gas exchange. Recently, one of the authors (T.T.) and his colleagues examined this hypothesis through quantification of the oxygen-supplying ability of viviparous dogfishes (*Squalus* spp.)^6^. They used a physical model to estimate the rate of oxygen diffusion through the uterine villi and concluded that the oxygen supply from uterine villi cannot support the embryonic oxygen demand. Previous studies have showed that dogfish periodically exchange uterine fluid with external seawater through the cervix in late gestation^7,8^. Thus, it was suspected that the seawater introduced from the external environment is the main source of embryonic oxygen^6^. However, such studies have thus far been restricted to dogfish, and the large diversity in the uterine surface structure among elasmobranchs prevents direct generalization with other taxa.

The present study evaluates the oxygen-supplying ability of the white shark (*Carcharodon carcharias*), the largest piscivorous shark. To the best of our knowledge, this is the second aplacental viviparous elasmobranch for which the oxygen-supplying ability has been estimated until date. This species produces 2 to 10 embryos through single gestation. The embryo grows to a total length of 1.6 m in utero by consuming lipid-rich uterine “milk” and unfertilized eggs^9–11^. Lamniform sharks, including the white shark, are known to have numerous lamellae covering the entire inner surface of the uterus. This structure is hypothesised to ensure oxygenation of the uterine fluid^11–13^, although this has never been tested quantitatively. Here, we describe the detailed morphology of the uterine surface structure of white shark for the first time and evaluate its ability to supply oxygen to the embryo.

## Results

### Structure of uterine surface

**Figure 1:**
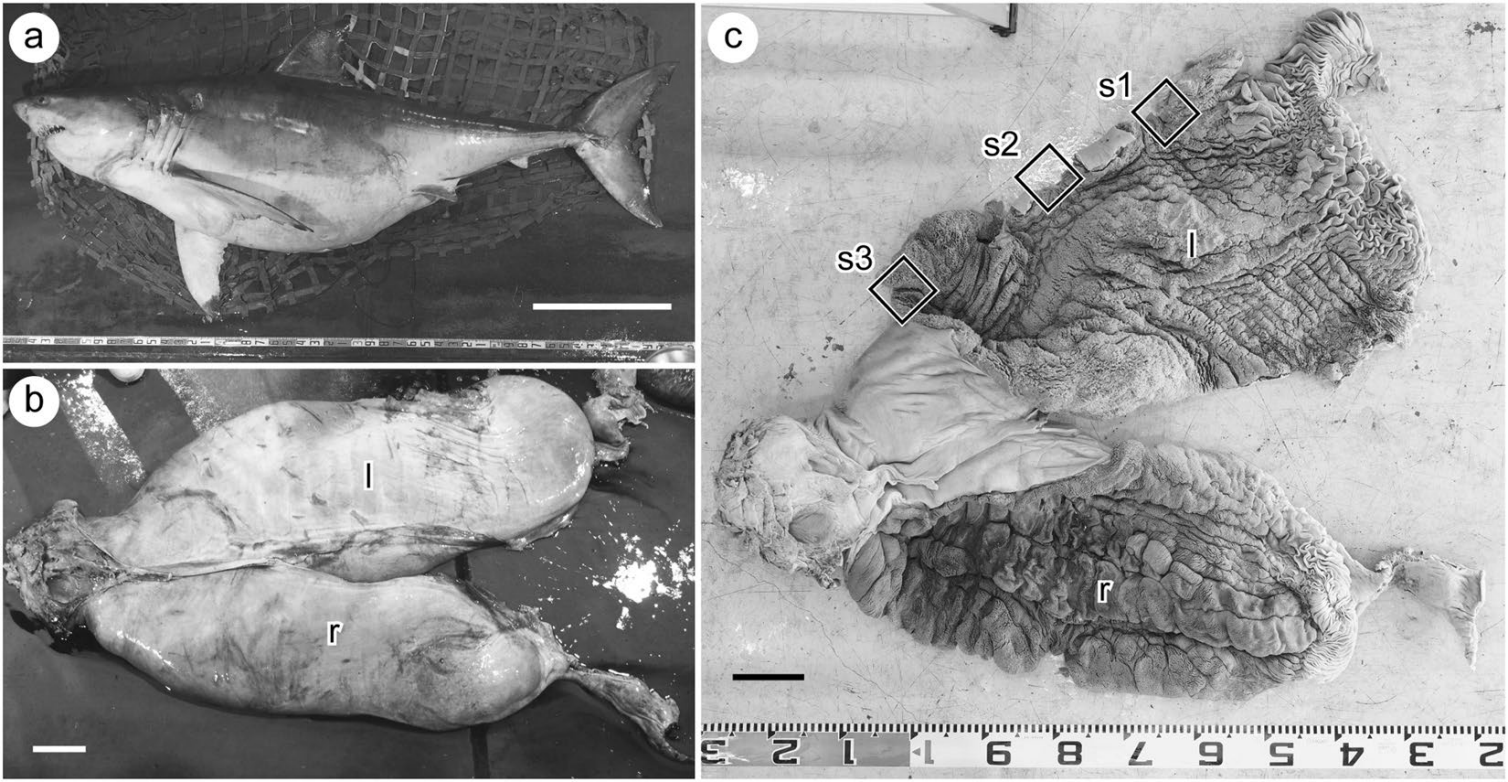
Uterine specimen of white shark examined in this study (OCF-P 03062). (**a**) The pregnant adult female white shark from which the uterus was extracted. (**b**) Right (r) and left (l) uteri of the white shark. (**c**) Dissected uteri. Numerous lamellae cover the entire inner surface of the uterus. Black squares highlight the three sampling locations for uterine tissue (sites 1–3). Scale bar = 1 m in (a) and 10 cm in (**b**) and (**c**).

**Figure 2:**
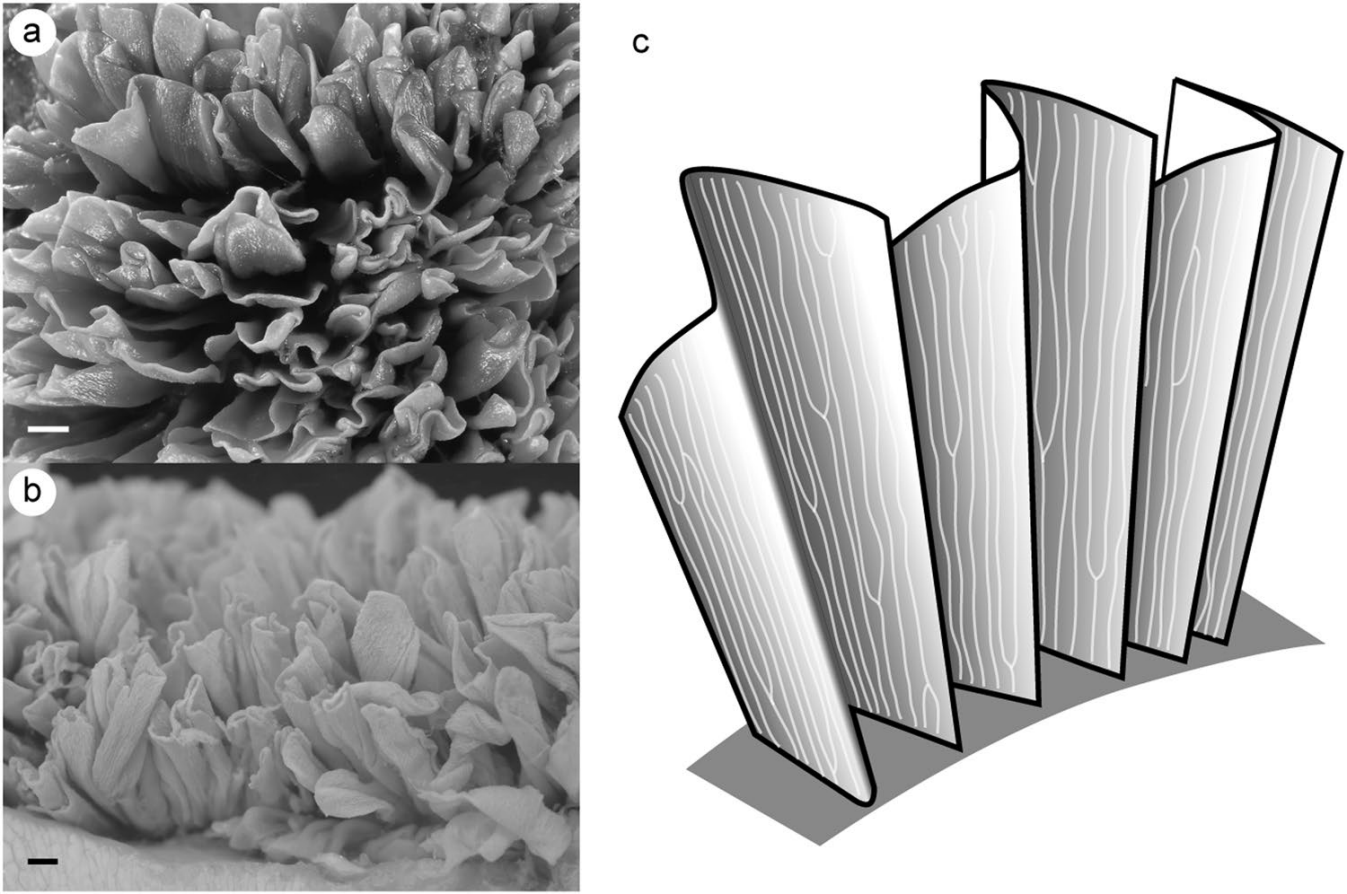
Uterine lamellae of white shark. (**a**) Above view of the uterine lamellae. (**b**) Lateral view of the uterine lamellae. (**c**) Schematic representation of the uterine lamella folded in accordion-like fashion. Scale bar = 1 mm in (**a**) and (**b**).

**Figure 3:**
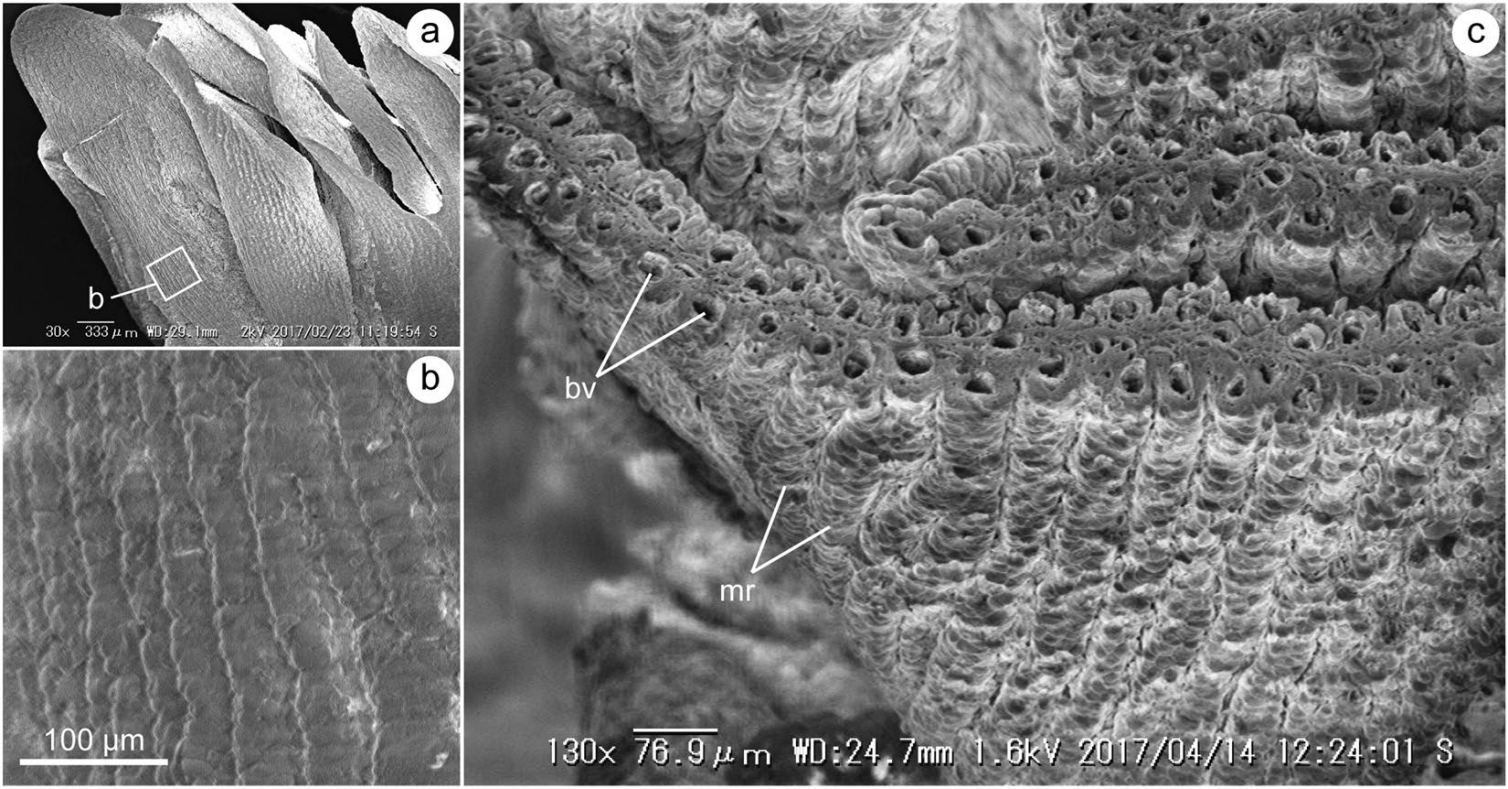
Scanning electron microscopy (SEM) images of the uterine wall. (**a**) Entire view of the uterine lamellae. (**b**) Close-up view of the white box in (**a**) showing the micro-ridges developed on the lamellar surface. (**c**) Cross section of the uterine lamellae showing a single blood vessel (bv) in each micro-ridge (mr).

**Figure 4:**
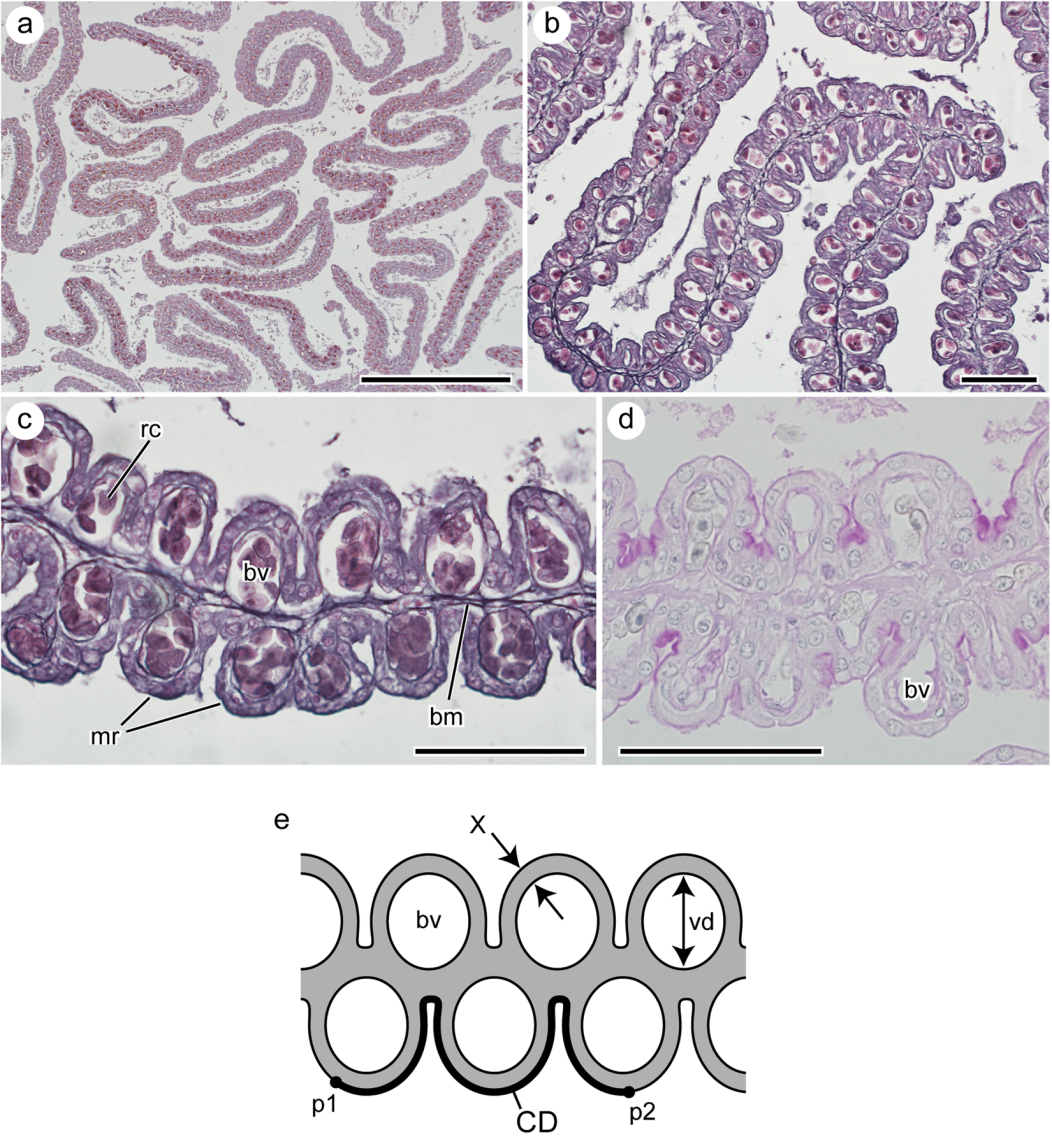
Histological thin section of the uterine lamellae. (**a**) Cross section of the uterine lamellae cut through the mid-half of lamellar height. (**b**) Close-up view of the uterine lamellae. (**c**) Close-up view of a part of a single lamella showing a blood vessel (bv) running through each micro-ridge (mr). (**d**) PAS-stained uterine surface. (**e**) Schematic representation of the cross section of uterine lamellae showing the locations of morphometric measurements. Abbreviations: bm, basement membrane; vd, blood vessel diameter; rc, red blood cell; CD, curved distance along the lamellar surface between two points (p1 and p2; linear distance between p1 and p2 = 200 µm); X, thickness of the diffusion barrier. Scale bars = 1 mm in (**a**) and 100 µm in (**b**), (**c**) and (**d**).

Numerous uterine lamellae were observed on the entire surface of the uterine wall (Fig. 1). Each lamella was tightly folded in an accordion-like fashion (Fig. 2). Numerous micro-ridges ran parallel toward the free margin of the lamellae on both sides of the uterine lamellae (Fig. 3). Each micro-ridge contained a single blood vessel (Fig. 3c, Fig. 4). The average diameter of the blood vessel was 33.9 ± 4.4 (SD) µm. The surface of the lamella consists of a single layer of epithelial cells, and these cells are supported by the basement membrane (Fig. 4c). The thickness of the diffusion barrier, which is defined as the distance between the wall of the blood vessel and the surface of lamellae, was 11.5 ± 2.1 (SD) µm. The lamellar surfaces were periodic acid-Schiff (PAS)-negative in most parts, but were weakly stained at the basal-most portion of the micro-ridges (Fig. 4d).

### Oxygen-diffusing capacity of uterine lamellae

Morphometric measurements for calculating the surface area and oxygen-diffusing capacity are listed in Table 1. The surface area of the whole uterus was estimated to be 21.6 ± 6.6 (SD) m^2^; the oxygen-diffusing capacity of the whole uterus was estimated to be 24.6 ± 8.8 (SD) ×10^4^ nmol·min^−1^·torr^−1^; and the oxygen-diffusing capacity of 1 cm^2^ of the uterine wall was estimated to be 63.6 ± 22.7 (SD) nmol·min^−1^·torr^−1^. There was no significant difference in the oxygen-diffusing capacity among anterior, intermediate, and posterior portion of the uterus (ANOVA, p > 0.05).

**Table 1:** Morphometric measurements (±SD) of the uterine lamellae, and estimated oxygen-diffusion capacity of white shark uterus.

Sampling locations	Projected area oflamellae S (cm^2^)^*1^	Surface increase because of the presence of microridges α	Thickness of the diffusion barrier X (μm)	Oxygen-diffusion capacity D (nmol × min^−1^ × torr^−1^)^*1,2^
Anterior (s1)	16.1 (±0.4)	1.96 (±0.50)	11.9 (±1.9)	69.5 (±21.0)
Intermediate (s2)	12.1 (±2.5)	2.17 (±0.38)	11.8 (±2.0)	58.3 (±18.6)
Posterior (s3)	11.1 (±1.8)	2.25 (±0.49)	10.8 (±2.3)	60.6 (±20.9)
Total	13.1 (±2.8)	2.13 (±0.47)	11.5 (±2.1)	63.6 (±22.7)

^*1^Values for 1 cm^2^ of the uterine wall

^*2^D = K × 2 × α × S/X, K = 1.31 × 10^−3^ nmol × cm^−1^ × min^−1^ × torr^−1^ (see text for details).

## Discussion

The present study provides the first quantitative evidence to suggest the high oxygen-supplying ability of lamniform shark uterus. We found three morphological features, which greatly increase the surface area of the white shark uterus: (1) uterine lamellae developed perpendicular to the uterine wall, (2) uterine lamellae folded in an accordion-like fashion, and (3) numerous micro-ridges developed on the lamellar surface. These features result in the increase in uterine surface to 56 times compared to the uterus that has a smooth surface. Similar uterine lamellae were also reported in *Cetorhinus maximum* ^14^, *Isurus oxyrinchus* ^12^, and *Carcharias taurus* ^13^, suggesting that they are shared among Lamniformes. The surface projections (e.g., uterine villi) have been observed in many aplacental elasmobranchs such as squaloid sharks (*Centrophorus*, *Centroscyllium*, *Centroscymnus*, *Deania*, *Etmopterus*, *Pristiophorus*, and *Squalus*) and stingrays (*Dasyatis*, *Myliobatis*, *Gymnura*, *Rhinoptera*, *Urolophus*, and *Manta*)^3,15–20^. However, these structures differ from those of lamniform sharks in possessing a simple filamentous morphology.

The oxygen-diffusing capacity of white shark uterus is greater than that of viviparous dogfish. The surface area of the white shark uterus is about 12 to 20 times greater than that of dogfish for the same tissue size (mean ± SD = 1.97 ± 0.19 and 1.25 ± 0.42 cm^2^ per 1-cm-squared uterine tissue in *Squalus* cf. *mitsukurii* and *S*. *cubensis*, respectively^6^). Moreover, the gas-diffusing barrier of the uterine wall of white shark is about one-tenth of that of dogfish (mean ± SD = 0.11 ± 0.02 and 0.11 ± 0.01 mm in *S*.cf. *mitsukurii* and *S*. *cubensis*, respectively^6^). Using these values, the oxygen-diffusing capacity of white shark is estimated to be about 250–400 times greater than that of dogfish for the same tissue size.

The present study sheds light on the diversity of the mechanism of oxygen acquisition in viviparous elasmobranchs. It has been suspected that the dogfish embryo uses uterine seawater from external environment as a major source of oxygen^6^. However, this mechanism is unlikely to occur in white shark, because its cervix is quite narrow compared to the uterine size (maximum diameter of cervix was c.a. 1.5 cm) and is tightly closed. This condition is also seen in another gravid white shark specimen (OCF-P 03018) that was incidentally caught by local fishermen in Okinawa Prefecture, Japan, in 2016. The uterine fluid (>130 L) of this specimen was retained in the uterus for more than two days after its death, and no leak of uterine fluid through the cervix was seen even after the specimen was lifted on land (S.M., T.T., and K.S., unpublished data). Narrow and tightly closed cervix was also reported in lamnoid *Cetorhinus maximum* (Matthews, 1949). In addition, little or no water input from the external environment was supported by the chemical composition of uterine fluid of the white shark (OCF-P 03062). By using DRI-CHEM 7000 V (Fujifilm Co., Tokyo, Japan), Na^+^, K^+^, and Cl^−^ concentration of the uterine fluid was measured to be 402, 4.5, and 366 mEq/L, respectively, and all of these values were significantly lower than those of seawater. From these observations, we can hypothesize that the amount of water input through cervix, if any, is quite limited in white shark, and the embryonic oxygen is mainly supplied from the uterine wall.

The present study also revealed that the oxygen-diffusing capacity of white shark is comparable to that of fish gills. Oxygen-diffusing capacities of the gills vary among species reflecting their locomotor lifestyles: Demersal fishes generally have lower oxygen-diffusing capacity compared to fast-swimming pelagic fishes^21^. Based on our calculations, the oxygen-diffusing capacity of 1 cm^2^ gill tissue of benthic *Scyliorhinus stellaris* was 6.7 nmol·min^−1^·torr^−1^, whereas it was 82.3 nmol·min^−1^·torr^−1^ in pelagic *Isurus oxyrinchus*. This indicates that the oxygen-diffusing capacity of white shark uterus (63.6 nmol·min^−1^·torr^−1^) is nearly comparable to the value of the gills of *I*. *oxyrinchus*.

The uterine wall of white shark probably exchanges many kinds of molecules and materials with the uterine fluid but their contents and proportions may be drastically changed during gestation. Previous study showed that the uterine wall in early gestation includes many secretory cells that produce lipid-rich “milk”^11^. Our study showed that such milk secretion probably ceased in late gestation, because PAS-staining reaction to the uterine surface in late gestation is much weaker than that in early gestation (Fig. 4 in ref.^11^). On the other hand, micro-ridges of the uterine lamellae become more prominent, and the epithelium of uterine lamella is changed from double- to single-cell layered from early to late gestation (Fig. 4 in ref.^11^). These modifications result in the increase in surface area and decrease in the thickness of the diffusion barrier, both leading to increase in oxygen-diffusion capacity. Based on these observations, it is likely that major functions of the uterine wall involve both milk secretion and respiration in early gestation but are more specialized to respiration in late gestation. This phenomenon may be reflected by the abrupt increase in oxygen demand in large developing embryos, which was observed in oviparous species^22,23^.

## Materials and Methods

### White shark uterus

The uterine specimen was obtained from a single female white shark OCF-P 03062 (5.05 m in total length, TL), which was the bycatch of the set net in 2016 by local fishermen in Okinawa Prefecture, Japan (Fig. 1). The left uterus of the specimen included four late-term embryos (c.a. 100 cm TL), and the right uterus was empty. The description of the embryos was provided previously^11^. We took the samples of the uterine walls (c.a. 5 cm × 5 cm) from the anterior, intermediate, and posterior portions of the left uterus (sites s1–s3 in Fig. 1c) and fixed it in Bouin’s solution for histological study (see below). The rest of the uterine specimen was fixed in 10% formalin for further studies. All the specimens used in this study were preserved at Okinawa Churashima Research Center (Okinawa Churashima Foundation, Okinawa, Japan). The small sample size is a consequence of the scarcity in the availability of pregnant white shark specimens.

### Histological thin section

The tissue samples were sectioned at 7 µm almost parallel to the wall and were stained with Delafield’s hematoxylin and eosin (Fig. 4a–c). Some sections were stained with PAS staining kit (1.01646.0001; Merck KGaA) and counterstained with hematoxylin to confirm the presence/absence of milk secretion.

### Oxygen-diffusing capacity

We evaluated the oxygen-supplying ability of the uterine wall by using oxygen-diffusing capacity (D, nmol·min^−1^·torr^−1^). This index is widely used to quantify the efficiency of passage of gas molecules through the biological membrane, such as lung and fish gills^24^. Oxygen-diffusing capacity is defined as follows:

1 $${\rm{D}}={\rm{K}}\times {\rm{A}}/{\rm{X}}$$

where K is the Krogh’s diffusion coefficient, A (cm^2^) is the surface area of the uterine surface, and X (cm) is the thickness of the diffusion barrier. The value of K varies among tissues, but its value in a biological membrane is typically one-third the value in pure water^25^. Following a prior study of oxygen diffusion in skate external gill filaments^26^, we used the value of 1.31 × 10^−3^ nmol·cm^−1^·min^−1^·torr^−1^ for K^27^.

### Surface area and thickness of the diffusion barrier of the uterus

The tissue samples of the uterine wall (1 cm × 1 cm) were obtained from the anterior, intermediate, and posterior portion of the left uterus (sites s1–s3 in Fig. 1c). We collected five samples from each site. Then, we used a scapula to isolate all the uterine lamellae for each tissue and mounted them on slide grasses with Aqueous Mount-Quick (Daido Sangyo Co., Tokyo, Japan) (Fig. S1). All these lamellae were photographed using a digital microscope (BX53; Olympus Co., Tokyo, Japan) at Okinawa Churashima Research Center, and the projected areas of the lamellae were measured using ImageJ software (US National Institutes of Health, Bethesda, MD, USA).

The effect of the increase in surface caused by the micro-ridges was evaluated in the following sequence. First, the histological sections were photographed using a digital microscope (BX53; Olympus Co., Tokyo, Japan). Second, a pair of points located on the lamellar surface was set for these photographs, so that the linear distance between these points was 200 µm (p1 and p2 in Fig. 4d). Third, the curved distance (CD, µm) along the surface of the uterine wall between two points was measured using ImageJ. Fourth, CD/200 (µm/µm) was calculated to determine the effect of the increase in surface caused by the micro-ridges.

Assuming the cylindrical morphology of the uterus, we calculated the surface area of the whole uterus as follows:

2 $${\rm{A}}=2\times {\rm{\alpha }}\times {\rm{S}}\times {\rm{UD}}\times {\rm{UC}}$$

where A (cm^2^) is the surface area of the uterine wall, S (cm^2^) is total projected area of lamellae within 1-cm-squared tissue sample, α is the effect of the increase in surface caused by the micro-ridge on the lamellar surface, UD (cm) is the antero-posterior length of the uterus, and UC (cm) is the circumference of the uterus at the middle half of the uterus. Using a measuring tape, we measured UD and UC, which were 90 and 43 cm, respectively.

The thickness of the diffusion barrier (X) was measured from histological thin sections using the digital microscope (BX53; Olympus Co., Tokyo, Japan) (Fig. 4d). Thirty measurements were taken from each sampling site (sites s1–s3 in Fig. 1c), and the average was calculated for each site.

Notably, the isthmus region was not included in our physical calculation. Isthmus region, which is the anterior-most small region of the uterus, is characterized by the development of large ridges on the surface (Fig. S2a). This feature is presumed to allow enhanced oxygen supply in this region^12,13^. However, this notion is not supported by the following two lines of evidence. (1) Contribution of large ledges to increase in surface-area is relatively small; it is approximately four times over that of the flat surface (=1.4 [number of large ridges per cm of the isthmus region] × 1.4 cm [approximate height of the large ridges] × 2 [the number of sides of each large ridge]). (2) Microstructure suggests that the ability of oxygen supply of the isthmus region is lower than that of the other regions. The height of uterine lamellae is approximately one-tenth of the other regions, and the diffusion barrier is approximately 2.3 times thicker than that of the other regions (height of uterine lamellae = 0.54 ± 0.07 [SD] mm; thickness of diffusion barrier = 24.9 ± 3.6 [SD] µm; Fig. S2b–d). Based on these observations, the amount of oxygen supply in this region is probably smaller than that in other regions, suggesting that the exclusion of the isthmus region may have little effect on our conclusions.

### Oxygen-diffusing capacity of fish gills

The oxygen-diffusing capacity of gills was calculated for two elasmobranch species, *Isurus oxyrinchus* and *Scyliorhinus stellaris*. Based on the geometrical model for fish gills in ref.^28^, the oxygen-diffusing capacity of single gill filament (D_gill_) was calculated as follows:

3 $${{\rm{D}}}_{{\rm{gill}}}={\rm{K}}\times (2{\rm{n}}\times {\rm{LW}}\times {\rm{LH}})/{{\rm{X}}}_{{\rm{gill}}}$$

where K is Krogh’s diffusion coefficient, n is average number of half-side of secondary lamella of a single filament, LW (cm) is the average width of secondary lamella, LH (cm) is the average height of secondary lamella, and X_gill_ (cm) is the thickness of the diffusion barrier of the gills. D_gill_ was divided by the average projected area of a single gill filament (=average length of gill filament [cm] × average width of gill filament [cm]) for estimating the oxygen-diffusing capacity of 1 cm^2^ gill tissues. All morphometric data of the gills used for this analysis were obtained from previous publications^21,29,30^. The value of 1.31 × 10^−3^ nmol·cm^−1^·min^−1^·torr^−1^ was used for K.

### Scanning electron microscopy

Scanning electron microscope (SEM) images were obtained for five tissue samples of the uterine wall (approximately 3 mm × 3 mm) using a VE-8800 (Keyence Co., Osaka, Japan) at 1–3 kV at the Okinawa Churaumi Aquarium (Okinawa, Japan) from each of the three sites. Before SEM imaging, the tissue samples were dehydrated through a graded series of ethanol (70, 80, 90, 95, and 99.5%) and 100% *tert*-butyl alcohol and were freeze-dried using EYELA FDU-1200 (Tokyo Rikakikai Co., Tokyo, Japan) at Okinawa Churaumi Aquarium.

## Electronic supplementary material

Supplementary Materials
