# Supplementary material for: Live-bearing without placenta: Physical estimation indicates the high oxygen-supplying ability of white shark uterus to the embryo
Source: Sci Rep. 2017 Sep 18;7:11744. doi: 10.1038/s41598-017-11973-9 (PMC5603572; doi:10.1038/s41598-017-11973-9)
Supplement: Supplementary file 1 — Supplementary Materials [file 41598_2017_11973_MOESM1_ESM.doc]

**Supplementary Materials**

**for**

**Live-bearing without placenta: Physical estimation indicates**

**the high oxygen-supplying ability of white shark uterus to the embryo**

Taketeru Tomita^1,2*^, Ryo Nozu^1,2^, Masaru Nakamura^1^, Shohei Matsuzaki^2^,

Kei Miyamoto^1,2^, & Keiichi Sato^2^

^1^*Zoological Laboratory*, *Okinawa Churashima Research Center*, *888 Ishikawa, Motobu-cho*, *Okinawa 905-0206*, *Japan*.

^2^*Okinawa Churaumi Aquarium*, *424 Ishikawa*, *Motobu-cho*, *Okinawa 905-0206*, *Japan.*

^*^Author for correspondence (email: [t-tomita@okichura.jp](mailto:t-tomita@okichura.jp))


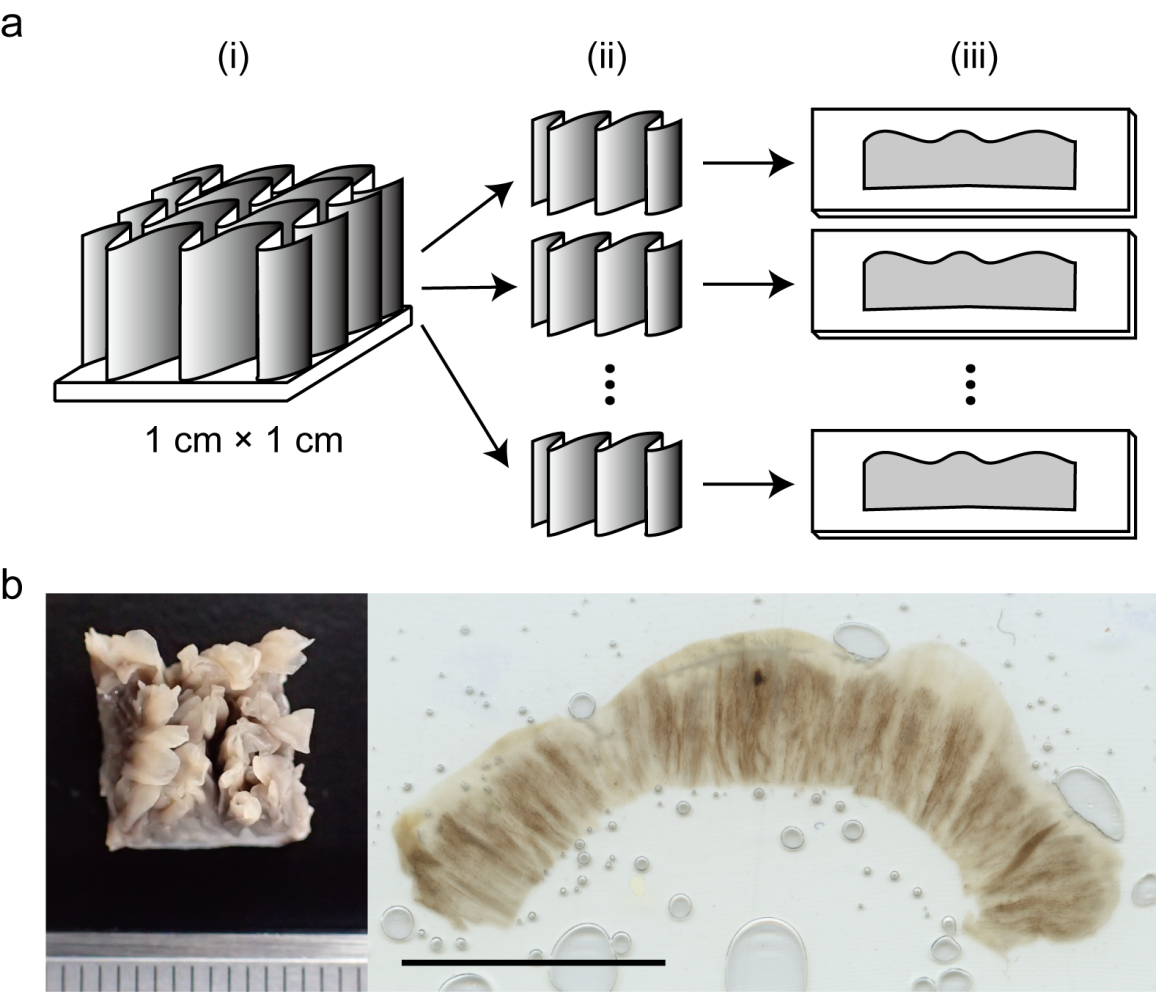


**Fig. S1** (**a**) Schematic diagram showing the process for measuring the total projected area of uterine lamellae for a 1 cm^2^ sample of uterine wall. A 1 cm^2^ tissue sample was obtained from a white shark uterus (a-i). All the uterine lamellae were separated (a-ii). Separated uterine lamellae were extended, and mounted on the glass slides (a-iii). Projected areas were measured for each uterine lamella for each glass slide, and the sum of all slides was calculated. (**b**) Upper view of 1 cm^2^ uterine tissue (left), and one example of uterine lamella extended and mounted on a glass slide (right). Scale bar = 1 cm.


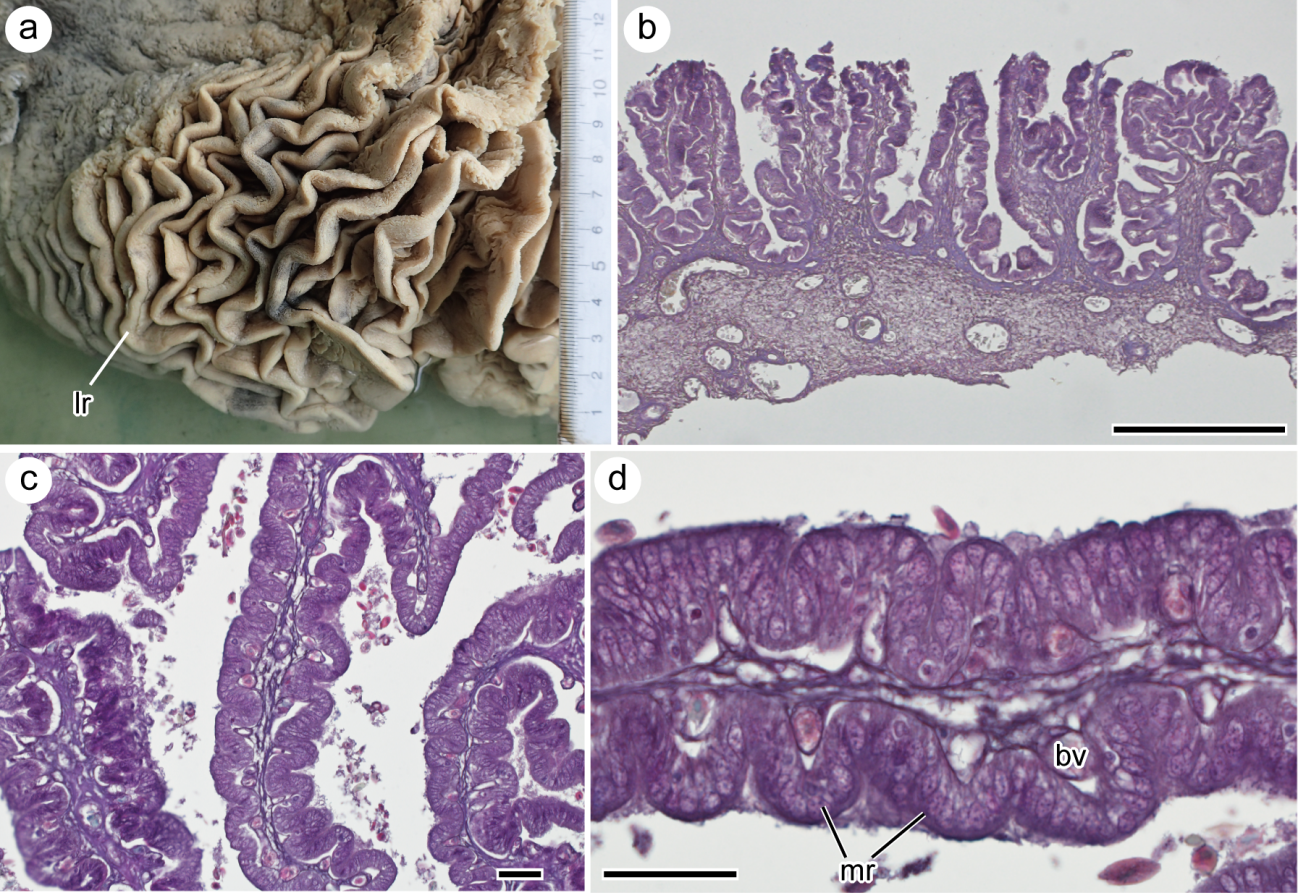


**Fig. S2** (**a**) Isthmus region of the uterus. Large ridges (lr) are present on its surface. (**b**) A vertical histological cross-section of a uterine lamella developed on the surface of large ridges. (**c**) A horizontal cross-section of a uterine lamella. (**d**) Magnified view of a horizontal cross-section of a uterine lamella showing the micro-ridges (mr) developed on its surface. A single blood vessel (bv) is included in each micro-ridge. The 7-µm thick tissue sample cross-sections were stained with Delafield’s hematoxylin and eosin. Scale bar = 0.5 mm in (**b**), and 50 µm in (**c**) and (**d**).
